# Mpox-Related Knowledge, Risk Perception, and Vaccination Willingness Among University Students in Aktobe, Kazakhstan: A Cross-Sectional Study

**DOI:** 10.3390/vaccines14060504

**Published:** 2026-06-03

**Authors:** Dilnaz Konbayeva, Lespek Kutumbetov, Balzhan Myrzakhmetova, Olga Chervyakova, Kuandyk Zhugunissov, Askhat Myngbay, Gulnar Altayeva, Saida Askatkyzy, Togzhan Nurdauletova, Gulmira Abulgazimova, Nadezhda Burambayeva, Arman Issimov

**Affiliations:** 1 Department of Biology, K. Zhubanov Aktobe Regional University, Aktobe 030000, Kazakhstanaskhat.myngbay@zhubanov.edu.kz (A.M.); 2 Research Institute for Biological Safety Problems, Gvardeiskiy 080423, Kazakhstan; 3 Department of Life Sciences, K. Zhubanov Aktobe Regional University, Aktobe 030000, Kazakhstan; 4 Institute of Animal Science and Veterinary Medicine, Saken Seifullin Kazakh Agrotechnical University, Astana 010000, Kazakhstan; 5 Department of Zootechnology, Genetics and Breeding, Toraighyrov University, Pavlodar 140000, Kazakhstan

**Keywords:** Mpox, monkeypox, health literacy, vaccine hesitancy, vaccination willingness, university students, risk perception, Kazakhstan

## Abstract

Background: Mpox is a re-emerging viral zoonotic disease that remains relevant for public health preparedness, risk communication, and vaccination literacy. University students are an important population for infectious disease communication because they are socially active, digitally connected, and may act as knowledge multipliers. This study assessed Mpox-related knowledge, risk perception, preventive practices, and willingness to receive Mpox vaccination among university students in Aktobe, Kazakhstan, and identified independent predictors of adequate knowledge and vaccination willingness. Methods: A cross-sectional questionnaire-based survey was conducted among students from three universities. The questionnaire collected socio-demographic characteristics, Mpox-related knowledge, information sources, attitudes, preventive practices, perceived risk, and vaccination willingness. Knowledge was summarized using a three-item score; adequate knowledge was defined as a score of at least 2 out of 3. Two multivariable logistic regression models were fitted: one for adequate Mpox-related knowledge and one for willingness to receive Mpox vaccination. Results: The final descriptive dataset included 482 respondents. Most participants were female (66.8%), from urban areas (80.5%), and aged 17–18 years (61.6%). Only 217 students (45.0%) had previously heard about Mpox, 193 (40.0%) correctly identified rash as the main symptom, and 179 (37.1%) knew that vaccination against Mpox exists. Adequate knowledge was observed in 201 students (41.7%), while only 150 students (31.1%) were willing to receive Mpox vaccination. In the multivariable model, adequate knowledge was independently associated with studying at Marat Ospanov West Kazakhstan Medical University (aOR = 5.66; 95% CI: 2.95–10.84), use of medical websites as an information source (aOR = 1.71; 95% CI: 1.09–2.69), and following infectious disease news (aOR = 2.81; 95% CI: 1.76–4.48). Vaccination willingness was independently associated with considering Mpox a dangerous infectious disease (aOR = 2.04; 95% CI: 1.11–3.77) and perceiving Mpox as a threat to Kazakhstan (aOR = 2.16; 95% CI: 1.30–3.59). Conclusions: Mpox-related knowledge among university students in Aktobe was limited, while vaccination willingness remained low despite moderate perceived risk. Reliable information exposure improved knowledge, whereas vaccination willingness was more strongly associated with perceived disease threat. These findings support university-based health literacy, vaccine literacy, and risk communication interventions aimed at improving preparedness for emerging infectious diseases.

## 1. Introduction

Mpox is a viral disease caused by monkeypox virus, a member of the Orthopoxvirus genus. The disease is commonly characterized by skin rash or mucosal lesions, fever, headache, myalgia, low energy, and lymphadenopathy. Transmission may occur through close physical contact with an infected person, exposure to contaminated materials, respiratory particles during close contact, or contact with infected animals [1,2,3]. Although Mpox was historically concentrated in endemic areas of Central and West Africa, the 2022 multi-country outbreak demonstrated its potential for sustained international spread and changed the epidemiological profile of the disease [4,5]. Global Mpox dynamics continue to require public health attention. The World Health Organization (WHO) reported that Mpox surveillance, laboratory capacity, infection prevention and control, vaccination of people at risk, locally relevant risk communication, and community engagement remain important components of preparedness and response [1,4]. Clinically relevant prevention depends on early recognition, avoidance of close contact with symptomatic individuals, appropriate infection-control measures, and vaccination of eligible or at-risk groups according to national recommendations [6,7].

University students represent an important population for infectious disease preparedness and health communication [8]. They are highly mobile, socially active, and frequent users of digital information platforms [9]. Students may also function as knowledge multipliers by sharing health information with peers, families, and communities [10]. However, digital connectedness may increase exposure to incomplete, conflicting, or misleading health information, potentially contributing to uncertainty, misconceptions, and vaccine hesitancy. Assessment of knowledge, attitudes, and practices (KAP) among students can therefore provide useful evidence for university-based health literacy and risk communication interventions [11,12,13,14,15]. Previous KAP studies conducted among university students, healthcare students, healthcare personnel, and the general population have shown heterogeneous levels of Mpox-related knowledge, with important gaps in transmission routes, clinical recognition, prevention, and vaccination awareness [14,16,17,18,19]. The relationship between knowledge and vaccination behavior is complex. Factual knowledge alone may not be sufficient to ensure willingness to receive vaccination because vaccine-related decisions are shaped by risk perception, trust, perceived benefits, confidence, and broader psychological antecedents of vaccination [20,21,22,23,24]. This distinction is particularly important for emerging infections such as Mpox, where students may recognize general infectious disease risks but remain uncertain about disease-specific vaccination recommendations.

A Kazakhstan-based healthcare study of medical and health-program students showed that general, vaccination, navigational, and digital health literacy are important competencies for future health professionals [12]. In the context of Mpox, these competencies are relevant not only for medical students but also for non-medical students who may rely heavily on digital sources for information about emerging infectious diseases [25].

In Kazakhstan, published evidence on Mpox-related awareness, risk perception, and vaccination willingness among university students remains limited. Aktobe is a major educational and transport hub in West Kazakhstan, with multiple universities and a mobile student population. Understanding student awareness in this setting can help guide targeted health education, risk communication, and preparedness activities for emerging infections. Therefore, this study aimed to assess Mpox-related knowledge, risk perception, preventive practices, and willingness to receive Mpox vaccination among university students in Aktobe, Kazakhstan, and to identify independent predictors of adequate knowledge and vaccination willingness.

## 2. Materials and Methods

### 2.1. Study Design

This cross-sectional questionnaire-based KAP study was conducted among university students in Aktobe, Kazakhstan. The reporting of this observational study was guided by the STROBE recommendations for cross-sectional studies and the ChecKAP recommendations for reporting KAP studies [26,27].

Aktobe is one of the major educational and transport centers in West Kazakhstan. Data collection was carried out between March and September 2025. The study included students from K. Zhubanov Aktobe Regional University, Marat Ospanov West Kazakhstan Medical University, and Baishev University. Including both medical and non-medical universities allowed comparison of Mpox-related knowledge and preventive attitudes across different educational profiles.

### 2.2. Participants and Sample Size

The target population consisted of students enrolled in the participating universities. Participation was voluntary and anonymous. Students were eligible if they were enrolled at one of the participating universities and agreed to complete the questionnaire. Students who were not enrolled at the participating universities or did not agree to participate were excluded. The minimum required sample size was estimated using the Cochran formula for cross-sectional surveys, assuming a 95% confidence level, a 5% margin of error, and a 50% response distribution, which produced a minimum of 384 respondents. To increase precision and allow subgroup analysis, the survey aimed to recruit more than the minimum required number. The final descriptive dataset used for this revised analysis included 482 valid responses.

### 2.3. Questionnaire

A structured questionnaire was developed to assess socio-demographic characteristics, information sources, Mpox-related knowledge, attitudes, risk perception, preventive practices, and willingness to receive Mpox vaccination (Table S1). The questionnaire content was informed by previously published Mpox KAP studies among university students, healthcare students, healthcare personnel, and the general population [17,18,28,29]. The questionnaire was as a paper-based, self-administered survey to students from the participating universities between March and September 2025. Before completing the questionnaire, participants were informed about the purpose of the study, the voluntary nature of participation, and the anonymous and confidential handling of responses. No personally identifiable information was collected, and completion of the questionnaire was considered an indication of consent. The questionnaire consisted of closed-ended items. Socio-demographic variables included age, sex, university, academic year, faculty, and place of residence. Knowledge items assessed previous awareness of Mpox, recognition of rash as a main symptom, and knowledge of vaccine availability. Attitude and risk-perception items assessed whether respondents considered Mpox a dangerous infectious disease, whether they would worry if Mpox were reported at the university, and whether they perceived Mpox as a threat to Kazakhstan. Practice items assessed personal hygiene in public places, following infectious disease news, and prompt medical consultation if symptoms appeared. Information sources included social networks, medical websites, teachers, friends, and television; multiple responses were allowed.

### 2.4. Outcome Definitions

A composite Mpox-related knowledge score was calculated using three knowledge items available in the dataset: previous awareness of Mpox, correct identification of rash as a main symptom, and awareness that a vaccine against Mpox exists. Similar to previous KAP studies, correct responses were assigned one point, while incorrect and “don’t know” responses were assigned zero points [16,17,30]. The score ranged from 0 to 3. Adequate knowledge was defined as a score of at least 2 out of 3, corresponding to a threshold of at least 60%. Inadequate knowledge was defined as a score of 0 or 1. Willingness to receive Mpox vaccination was coded as a binary outcome: “yes” responses were coded as willing to vaccinate, while “no” and “don’t know” responses were coded as not willing or uncertain.

### 2.5. Statistical Analysis

The data were collected into a Microsoft Excel spreadsheet (Table S2), edited, and processed using the Python programming language (Jupyter Notebook, version 3.9.7). Categorical variables were presented as frequencies and percentages. Age was summarized as mean and standard deviation (SD). Variables included in the multivariable logistic regression models were selected a priori based on the study objectives, questionnaire domains, and conceptual relevance to each outcome, rather than by automated stepwise selection or solely by univariate statistical significance. Two multivariable logistic regression models were constructed. The first model assessed independent predictors of adequate Mpox-related knowledge. Predictors included sex, age, residence, university, academic year, information sources, and following infectious disease news. The second model assessed independent predictors of willingness to receive Mpox vaccination. Predictors included sex, age, residence, university, academic year, knowledge score, perceived disease danger, concern about a university-level case, perceived threat to Kazakhstan, following infectious disease news, and selected information sources. Adjusted odds ratios (aORs), 95% confidence intervals (CIs), and *p*-values were reported. Statistical significance was set at *p* < 0.05.

### 2.6. Ethics Statement

This study was conducted in accordance with the principles of voluntary participation, anonymity, and confidentiality. Participants were informed about the purpose of the survey before completing the questionnaire, and completion of the questionnaire was considered an indication of consent. No personally identifiable information was collected. Ethical approval was obtained from the Human Ethics Committee of S.D. Asfendiyarov Kazakh National Medical University (approval number: 03-090125). All procedures were carried out in compliance with the ethical principles outlined in the Declaration of Helsinki (2013), with full respect for participants’ rights.

## 3. Results

### 3.1. Socio-Demographic Characteristics

A total of 482 students were included in the descriptive analysis (Table 1). Female respondents accounted for 66.8% (322/482), while male respondents accounted for 33.2% (160/482). Age data were available for 481 respondents; age ranged from 17 to 29 years, with a mean of 18.80 ± 1.88 years. Most respondents were from K. Zhubanov Aktobe Regional University (58.1%), followed by Baishev University (23.9%) and Marat Ospanov West Kazakhstan Medical University (18.0%). Most students lived in urban areas (80.5%), and first-year students represented the largest academic group (51.7%).

### 3.2. Mpox-Related Knowledge

Prior awareness of Mpox was limited. Overall, 217 students (45.0%) had heard about Mpox before the survey, while 262 (54.4%) had not and three responses were missing (Table 2). When asked about the main symptom of Mpox, 193 students (40.0%) correctly selected rash, whereas 228 (47.3%) answered “don’t know”. Awareness of vaccine availability was also limited: 179 respondents (37.1%) knew that a vaccine against Mpox exists, while 259 (53.7%) answered “don’t know”. Based on the three-item knowledge score, 201 respondents (41.7%) were classified as having adequate knowledge.

As a supplementary descriptive analysis, adequate Mpox-related knowledge was stratified by university type. Adequate knowledge was observed in 69 of 87 medical university students (79.3%) and in 132 of 395 non-medical university students (33.4%), indicating a higher prevalence of adequate knowledge among students from the medical university (Table S3).

### 3.3. Risk Perception, Vaccination Willingness, and Preventive Practices

Risk perception was higher than factual knowledge. Most respondents considered Mpox a dangerous infectious disease (62.4%) and would be worried if Mpox were reported at their university (70.1%) (Table 3). However, only 150 students (31.1%) were willing to receive Mpox vaccination, while 227 (47.1%) were uncertain and 105 (21.8%) were unwilling. Preventive practices were generally favorable: 407 respondents (84.4%) reported following personal hygiene rules in public places, and 387 (80.3%) reported that they would seek medical attention promptly if symptoms appeared.

### 3.4. Sources of Information

Social networks were the most commonly reported information source, selected by 366 respondents (75.9%). Medical websites were reported by 234 respondents (48.5%), teachers by 154 (32.0%), friends by 81 (16.8%), and television by 63 (13.1%) (Table 4).

### 3.5. Predictors of Adequate Mpox-Related Knowledge

The multivariable logistic regression model for adequate Mpox-related knowledge showed good overall model fit (pseudo R^2^ = 0.179; likelihood ratio test *p* < 0.001) (Table 5). After adjustment, students from Marat Ospanov West Kazakhstan Medical University had significantly higher odds of adequate knowledge than students from K. Zhubanov Aktobe Regional University (aOR = 5.66; 95% CI: 2.95–10.84; *p* < 0.001). Students from Baishev University had lower odds of adequate knowledge than the reference university (aOR = 0.50; 95% CI: 0.26–0.96; *p* = 0.038). Use of medical websites as an information source (aOR = 1.71; 95% CI: 1.09–2.69; *p* = 0.019) and following infectious disease news (aOR = 2.81; 95% CI: 1.76–4.48; *p* < 0.001) were also independently associated with adequate knowledge.

### 3.6. Predictors of Willingness to Receive Mpox Vaccination

The multivariable logistic regression model for willingness to receive Mpox vaccination also showed good overall model fit (pseudo R^2^ = 0.170; likelihood ratio test *p* < 0.001) (Table 6). In adjusted analysis, vaccination willingness was independently associated with risk perception. Students who considered Mpox a dangerous infectious disease had higher odds of willingness to vaccinate (aOR = 2.04; 95% CI: 1.11–3.77; *p* = 0.022). Students who perceived Mpox as a threat to Kazakhstan also had higher odds of willingness to vaccinate (aOR = 2.16; 95% CI: 1.30–3.59; *p* = 0.003). Knowledge score showed a positive trend but did not reach conventional statistical significance after adjustment (aOR = 1.23 per one-point increase; 95% CI: 0.98–1.53; *p* = 0.073).

## 4. Discussion

This study provides evidence on Mpox-related knowledge, risk perception, preventive practices, information sources, and vaccination willingness among university students in Aktobe, Kazakhstan. The findings indicate that disease-specific knowledge was limited: fewer than half of respondents had previously heard about Mpox, only 40.0% correctly identified rash as a main symptom, and just 37.1% were aware that vaccination against Mpox exists. These gaps are important because accurate recognition of symptoms, transmission routes, and preventive options is essential for timely health-seeking behavior and effective risk communication [1,2,6,31].

The multivariable analysis strengthens the interpretation of the descriptive results. Adequate knowledge was independently associated with medical university affiliation, use of medical websites, and following infectious disease news. This suggests that structured educational exposure and reliable information sources are key determinants of Mpox-related knowledge. The higher odds of adequate knowledge among students from Marat Ospanov West Kazakhstan Medical University are consistent with the expected influence of medical and health-related training. The supplementary stratified analysis further showed that adequate knowledge was more frequent among medical university students than among non-medical university students. Therefore, the association between Marat Ospanov WKMU affiliation and adequate knowledge should be interpreted as an expected finding reflecting medical educational background and greater exposure to health-related information, rather than as an unexpected institutional effect. This finding aligns with previous evidence showing that healthcare students’ Mpox knowledge and health literacy may vary according to educational background, curriculum exposure, and access to reliable health information [12,17,19].

An important finding was the difference between predictors of knowledge and predictors of vaccination willingness. While knowledge was associated with educational and information-related variables, willingness to receive Mpox vaccination was independently associated with risk perception. Students who considered Mpox dangerous and those who perceived it as a threat to Kazakhstan were more likely to be willing to vaccinate. In contrast, knowledge score showed only a borderline association after adjustment. This finding is consistent with theoretical and empirical evidence showing that vaccination intention is shaped not only by factual knowledge but also by perceived susceptibility, perceived severity, trust, confidence, perceived benefits, and broader psychological antecedents of vaccination [20,32,33].

The low level of willingness to receive Mpox vaccination deserves particular attention. Only 31.1% of students were willing to be vaccinated, whereas nearly half were uncertain. This pattern suggests vaccine hesitancy or insufficient vaccine literacy rather than simple refusal. Studies among students and other populations show that vaccine-related decisions are influenced by perceived disease threat, confidence in vaccines, the clarity of recommendations, and the quality of information sources [16,32]. In the present study, uncertainty about vaccine availability was also high, indicating a need for clearer education about who should receive Mpox vaccination, when vaccination is recommended, and how vaccination fits into broader outbreak control strategies.

The dominance of social networks as an information source has practical implications. Although social media can rapidly disseminate health messages, it may also expose students to incomplete or misleading information. In our adjusted model, use of medical websites, but not social networks, remained independently associated with adequate knowledge. This distinction supports the need for universities to direct students toward verified health information sources, including official public health websites, university health services, and trained educators. These findings are consistent with health-literacy and infodemic-management literature emphasizing the importance of helping students access, appraise, and apply reliable health information [11,34].

The present findings have important implications for public health preparedness among university students. Although most respondents reported favorable general hygiene practices and moderate concern regarding Mpox, disease-specific knowledge and willingness to receive vaccination remained limited. Importantly, the regression analysis demonstrated that reliable information exposure was independently associated with better knowledge, whereas vaccination willingness was more strongly associated with perceived disease threat. A Kazakhstan-based study emphasized the importance of general, vaccination, navigational, and digital health literacy among medical and health-program students [12]. Our findings extend this concept to Mpox preparedness by showing that reliable information exposure and engagement with infectious disease news are important determinants of disease-specific knowledge among university students in Kazakhstan. These findings suggest that university-based interventions should strengthen vaccine literacy, trust in public health recommendations, and risk communication strategies rather than relying solely on general awareness campaigns [13]. Because social networks represented the dominant information source in this study, universities should prioritize dissemination of concise, evidence-based information through official digital platforms. This approach is particularly important in the context of digital misinformation and “infodemics,” which may contribute to uncertainty and vaccine hesitancy among students [34]. Previous research among vaccine-hesitant college students also emphasized the importance of improving students’ ability to critically evaluate online health information and identify reliable sources [11]. Future studies should evaluate the effectiveness of such interventions and further explore behavioral determinants of vaccine hesitancy and risk perception among university students.

This study has several limitations. First, the cross-sectional design does not allow causal inference. Second, the survey relied on self-reported responses, which may be affected by recall bias or social desirability bias. Third, the knowledge score was based on three available knowledge items, which provides a concise but limited measure of Mpox-related knowledge. Future studies should use a more detailed validated knowledge scale covering etiology, transmission, clinical symptoms, risk groups, prevention, vaccination, and response to suspected infection. Additionally, although the sample included students from medical and non-medical universities, the distribution of faculties and academic years was uneven, with a large proportion of first-year students. In addition, the total number of eligible students in the participating faculties and the total number of students approached were not recorded; therefore, a formal participation rate could not be calculated. Because participation was voluntary and the sample was not probability-based, the findings may not be fully generalizable to all students at the participating universities, to all university students in Aktobe, or to university students in Kazakhstan more broadly. Finally, “no” and “don’t know” responses were combined for the vaccination-willingness regression model; this approach is useful for identifying willingness but does not distinguish refusal from uncertainty.

Overall, this study suggests a multilevel gap: limited factual knowledge, moderate perceived risk, favorable general hygiene practices, and low vaccination willingness. This pattern indicates that students may accept general preventive behaviors but remain uncertain about disease-specific interventions such as vaccination. Addressing this gap requires not only providing facts about Mpox but also strengthening trust, reducing uncertainty, and clarifying the role of vaccination in outbreak prevention.

## 5. Conclusions

Mpox-related knowledge among university students in Aktobe was limited, while vaccination willingness remained low despite moderate perceived risk. Reliable information exposure improved knowledge, whereas vaccination willingness was more strongly associated with perceived disease threat. These findings support university-based health literacy, vaccine literacy, and risk communication interventions aimed at improving preparedness for emerging infectious diseases.

## Figures and Tables

**Table 1:** Socio-demographic characteristics of respondents.

Variable	Category	n	%
Sex	Female	322	66.8
	Male	160	33.2
Residence	Urban/city	388	80.5
	Rural/district	94	19.5
Age group	17–18	297	61.6
	19–20	95	19.7
	21–23	77	16.0
	≥24	12	2.5
	Missing	1	0.2
University	K. Zhubanov Aktobe Regional University	280	58.1
	Marat Ospanov West Kazakhstan Medical University	87	18.0
	Baishev University	115	23.9
Academic year	First year	249	51.7
	Second year	140	29.0
	Third year	24	5.0
	Fourth year	29	6.0
	Master’s or combined response	40	8.3

**Table 2:** Students’ knowledge of Mpox symptoms and preventive measures (n = 482).

Question	Response	n	%
Have you heard about Mpox before?	Yes	217	45.0
	No	262	54.4
	Missing	3	0.6
Main symptom of Mpox	Rash	193	40.0
	Headache	32	6.6
	Diarrhea	29	6.0
	Do not know	228	47.3
Is there a vaccine against Mpox?	Yes	179	37.1
	No	44	9.1
	Do not know	259	53.7
Knowledge-score category	Adequate knowledge (score ≥ 2/3)	201	41.7
	Inadequate knowledge (score 0–1/3)	281	58.3

**Table 3:** Risk perception, vaccination willingness, and preventive practices regarding Mpox (n = 482).

Question	Yes, n (%)	No, n (%)	Do Not Know, n (%)	Missing, n (%)
Do you consider Mpox a dangerous infectious disease?	301 (62.4)	29 (6.0)	152 (31.5)	0 (0.0)
Would you worry if Mpox were reported at the university?	338 (70.1)	30 (6.2)	114 (23.7)	0 (0.0)
Are you willing to get vaccinated against Mpox?	150 (31.1)	105 (21.8)	227 (47.1)	0 (0.0)
Does Mpox pose a threat to Kazakhstan?	221 (45.9)	30 (6.2)	231 (47.9)	0 (0.0)
Do you follow personal hygiene rules in public places?	407 (84.4)	16 (3.3)	59 (12.2)	0 (0.0)
Do you follow news about infectious diseases?	305 (63.3)	72 (14.9)	104 (21.6)	1 (0.2)
If symptoms appear, will you seek medical attention promptly?	387 (80.3)	31 (6.4)	64 (13.3)	0 (0.0)

**Table 4:** Sources of information about infectious diseases/Mpox.

Information Source	n	%
Social networks	366	75.9
Medical websites	234	48.5
Teachers	154	32.0
Friends	81	16.8
Television	63	13.1

**Table 5:** Logistic regression analysis of factors associated with adequate Mpox-related knowledge (n = 481).

Predictor	Crude OR	95% CI	*p*-Value	Adjusted OR	95% CI	*p*-Value
Marat Ospanov WKMU (vs. K. Zhubanov ARU)	7.70	4.40–13.47	<0.001	5.66	2.95–10.84	<0.001
Follows infectious disease news	3.81	2.51–5.78	<0.001	2.81	1.76–4.48	<0.001
Medical websites as information source	2.63	1.81–3.82	<0.001	1.71	1.09–2.69	0.019
Baishev University (vs. K. Zhubanov ARU)	0.29	0.18–0.47	<0.001	0.50	0.26–0.96	0.038
Social networks as information source	2.01	1.28–3.15	0.002	1.63	0.96–2.76	0.071
Teachers as information source	1.17	0.79–1.72	0.432	0.72	0.45–1.17	0.184
Friends as information source	0.85	0.52–1.38	0.508	1.43	0.79–2.58	0.238
Television as information source	1.92	1.12–3.27	0.017	1.44	0.77–2.68	0.256
Rural residence (vs. urban)	0.84	0.53–1.34	0.472	0.77	0.44–1.35	0.364
Second-year students (vs. first-year)	1.47	0.99–2.18	0.057	1.23	0.73–2.05	0.439
Male sex (vs. female)	0.76	0.51–1.12	0.162	1.23	0.70–2.17	0.474
Third/fourth year or master’s students (vs. first-year)	0.79	0.49–1.26	0.318	1.31	0.56–3.06	0.527
Age, per one-year increase	0.99	0.90–1.09	0.902	1.00	0.85–1.18	0.987

Note: The descriptive dataset included 482 respondents; one respondent with missing age was excluded from the regression models, resulting in an analytic regression sample of 481 respondents.

**Table 6:** Logistic regression analysis of factors associated with willingness to receive Mpox vaccination (n = 481).

Predictor	Crude OR	95% CI	*p*-Value	Adjusted OR	95% CI	*p*-Value
Considers Mpox a threat to Kazakhstan	4.71	3.10–7.18	<0.001	2.16	1.30–3.59	0.003
Considers Mpox dangerous	5.31	3.21–8.76	<0.001	2.04	1.11–3.77	0.022
Knowledge score, per one-point increase	1.84	1.54–2.20	<0.001	1.23	0.98–1.53	0.073
Follows infectious disease news	3.52	2.21–5.60	<0.001	1.60	0.93–2.76	0.092
Medical websites as information source	2.44	1.64–3.64	<0.001	1.46	0.91–2.34	0.112
Marat Ospanov WKMU (vs. K. Zhubanov ARU)	2.66	1.65–4.27	<0.001	1.44	0.77–2.70	0.256
Would worry if Mpox reported at university	4.81	2.77–8.35	<0.001	1.43	0.72–2.82	0.306
Teachers as information source	1.64	1.09–2.45	0.017	1.27	0.79–2.05	0.325
Age, per one-year increase	1.07	0.97–1.19	0.168	1.08	0.92–1.28	0.345
Male sex (vs. female)	0.87	0.57–1.31	0.495	1.19	0.68–2.10	0.546
Second-year students (vs. first-year)	1.06	0.70–1.62	0.775	0.94	0.55–1.62	0.828
Baishev University (vs. K. Zhubanov ARU)	0.52	0.31–0.85	0.009	0.93	0.47–1.86	0.839
Rural residence (vs. urban)	0.88	0.53–1.44	0.598	0.96	0.54–1.71	0.884
Social networks as information source	1.24	0.78–1.97	0.365	0.97	0.57–1.65	0.905
Third/fourth year or master’s students (vs. first-year)	1.24	0.77–2.01	0.381	1.02	0.42–2.45	0.965

Note: The descriptive dataset included 482 respondents; one respondent with missing age was excluded from the regression models, resulting in an analytic regression sample of 481 respondents.

## Data Availability

The data supporting the findings of this study are available from the corresponding authors upon reasonable request.

## Supplementary Materials

The following supporting information can be downloaded at: https://www.mdpi.com/article/10.3390/vaccines14060504/s1, Table S1: Questionnaire; Table S2: Raw data; Table S3: Mpox-related knowledge stratified by university type; Supplementary File S1: Completed STROBE checklist for cross-sectional studies.
