# Supplementary material for: Mpox-Related Knowledge, Risk Perception, and Vaccination Willingness Among University Students in Aktobe, Kazakhstan: A Cross-Sectional Study
Source: Vaccines (Basel). 2026 Jun 3;14(6):504. doi: 10.3390/vaccines14060504 (PMC13308040; doi:10.3390/vaccines14060504)
Supplement: Supplementary file 1 [file vaccines-14-00504-s001.zip › Supplementary Materials/Supplementary_File_S1_STROBE.pdf]

Supplementary File S1. STROBE Checklist for Cross-Sectional Studies

Manuscript: Mpox-Related Knowledge, Risk Perception, and Vaccination Willingness among University Students in Aktobe, Kazakhstan: A Cross-Sectional Study

**Section/topic**	**Item**	**Reporting item**	**Location in manuscript**
Title and abstract	1a	Study design identified in the title or abstract.	Title; Abstract, Methods
Title and abstract	1b	Abstract summarizes the study aim, design, population, variables, main results, and conclusions.	Abstract, Background/Methods/Results/Conclusions
Introduction	2	Scientific background and rationale for the investigation are described.	Introduction, paragraphs 1-3
Introduction	3	Specific study objectives are stated.	Introduction, final paragraph
Methods	4	Key elements of the cross-sectional study design are presented early.	Section 2.2, Study Design
Methods	5	Study setting, location, participating institutions, and data collection period are reported.	Section 2.2, Study Design
Methods	6a	Eligibility criteria and sources/methods of participant selection are described.	Section 2.3, Participants and Sample Size
Methods	6b	Matching criteria are reported where applicable.	Not applicable; no matching was used
Methods	7	Outcomes, predictors, and relevant covariates are defined.	Sections 2.4-2.6
Methods	8	Data sources and measurement procedures are described for questionnaire variables.	Section 2.4, Questionnaire; Table S1
Methods	9	Potential sources of bias and relevant limitations are addressed.	Section 4, Discussion/Limitations
Methods	10	Study size calculation or rationale is provided.	Section 2.3, Participants and Sample Size
Methods	11	Handling of quantitative variables and grouping is explained.	Sections 2.5-2.6; Table 1
Methods	12a	Statistical methods and adjustment strategy are described.	Section 2.6, Statistical Analysis
Methods	12b	Subgroup or stratified analyses are described where applicable.	Section 3.2; Table S3
Methods	12c	Handling of missing data is described.	Section 2.6, Statistical Analysis; Tables 1-3; notes to Tables 5-6

Section/topic	Item	Reporting item	Location in manuscript
Methods	12d	Analytical approach accounts for the sampling strategy, where relevant.	Section 2.6; no complex sampling weights were applied
Methods	12e	Sensitivity analyses are reported if conducted.	Not applicable; no sensitivity analyses were performed
Results	13a	Numbers of participants included and analysed are reported.	Section 3.1; Tables 1, 5, and 6
Results	13b	Reasons for non-participation are given where available.	Not applicable; anonymous voluntary survey; non-participation reasons were not collected
Results	13c	Use of a participant flow diagram is considered.	Not applicable; participant numbers are reported in text and tables
Results	14a	Participant characteristics and relevant descriptive variables are presented.	Section 3.1; Table 1
Results	14b	Missing data are reported for variables with missing values.	Tables 1-3; notes to Tables 5-6
Results	14c	Follow-up time is reported for cohort studies.	Not applicable to this cross-sectional study
Results	15	Outcome data or summary outcome measures are reported.	Sections 3.2-3.3; Tables 2-3
Results	16a	Unadjusted and adjusted estimates, confidence intervals, and adjustment variables are reported.	Sections 3.5-3.6; Tables 5-6
Results	16b	Category boundaries are reported when continuous variables are categorized.	Table 1; Section 2.5
Results	16c	Relative-risk estimates are translated into absolute risks where relevant.	Not applicable; logistic regression odds ratios were reported
Results	17	Other analyses, including supplementary stratified analyses, are reported.	Section 3.2; Table S3
Discussion	18	Main findings are summarized with reference to study objectives.	Section 4, Discussion; Section 6, Conclusion
Discussion	19	Study limitations and possible sources/direction of bias are discussed.	Section 4, Limitations paragraph
Discussion	20	Interpretation considers objectives, limitations, related evidence, and public-health implications.	Section 4, Discussion
Discussion	21	Generalizability/external validity is considered.	Section 4, Limitations and public-health implications
Other information	22	Funding source is reported.	Funding statement

Abbreviations: STROBE, Strengthening the Reporting of Observational Studies in Epidemiology; WKMU, West Kazakhstan Medical University; ARU, Aktobe Regional University.
