# Supplementary material for: Mpox-Related Knowledge, Risk Perception, and Vaccination Willingness Among University Students in Aktobe, Kazakhstan: A Cross-Sectional Study
Source: Vaccines (Basel). 2026 Jun 3;14(6):504. doi: 10.3390/vaccines14060504 (PMC13308040; doi:10.3390/vaccines14060504)
Supplement: Supplementary file 1 [file vaccines-14-00504-s001.zip › Supplementary Materials/Table S1.pdf]

Socio-demographic Section

No	Questions	
1.	Age:	
2.	Educational institution:	
3.	Gender:	
4.	Faculty:	
5.	Year of study:	
6.	Residence:	

Knowledge

No	Questions	
7	Have you heard about monkeypox before? (Yes / No)	
8	Which of the following is a main symptom of monkeypox? (Rash / Headache / I do not know / Diarrhea)	
9	Is there a vaccine against monkeypox? (Yes / No / I do not know)	
10	Where did you get information about monkeypox? (Social media / Friends / Television / Medical websites / Teachers)	

Attitude

No	Questions	
11	I consider monkeypox a dangerous infectious disease. (Yes / No / I do not know)	
12	If monkeypox cases were reported at the university, I would be worried. (Yes / No / I do not know)	
13	Monkeypox poses a threat to Kazakhstan. (Yes / No / I do not know)	

Practice

No	Questions	
14	I am willing to get vaccinated against monkeypox. (Yes / No / I do not know)	
15	I follow personal hygiene rules in public places. (Yes / No / I do not know)	
16	I follow news about infectious diseases. (Yes / No / I do not know)	
17	If symptoms appear, I will immediately consult a doctor. (Yes / No / I do not know)	
