# Supplementary material for: Mpox-Related Knowledge, Risk Perception, and Vaccination Willingness Among University Students in Aktobe, Kazakhstan: A Cross-Sectional Study
Source: Vaccines (Basel). 2026 Jun 3;14(6):504. doi: 10.3390/vaccines14060504 (PMC13308040; doi:10.3390/vaccines14060504)
Supplement: Supplementary file 1 [file vaccines-14-00504-s001.zip › Supplementary Materials/Table S3.pdf]

Table S3. Mpox-related knowledge stratified by university type

University type	Universities included	Total, n	Adequate knowledge, n (%)	Inadequate knowledge, n (%)
Medical university	Marat Ospanov West Kazakhstan Medical University	87	69 (79.3)	18 (20.7)
Non-medical universities	K. Zhubanov Aktobe Regional University; Baishev University	395	132 (33.4)	263 (66.6)
Total	All participating universities	482	201 (41.7)	281 (58.3)
